# The Role of IL-23/Th17 Pathway in Patients with Primary Immune Thrombocytopenia

**DOI:** 10.1371/journal.pone.0117704

**Published:** 2015-01-26

**Authors:** Xin Ye, Lei Zhang, Hui Wang, Yan Chen, Weiwei Zhang, Rongrong Zhu, Chaoping Fang, Anmei Deng, Baohua Qian

**Affiliations:** 1 Department of Transfusion Medicine, Changhai Hospital, Second Military Medical University, 168 Changhai Road, Shanghai, 200433, China; 2 Department of Laboratory Diagnosis, Changhai Hospital, Second Military Medical University, 168 Changhai Road, Shanghai, 200433, China; Nippon Medical School Graduate School of Medicine, JAPAN

## Abstract

**Background:**

Primary immune thrombocytopenia (ITP) is an autoimmune bleeding disorder with an unclear etiology. This study aims to investigate the role of IL-23/Th17 pathway in patients with ITP.

**Method:**

The gene expressions of IL-17, IL-23 and their receptors in ITP patients and healthy controls were analyzed by quantitative real-time PCR. ELISA was used to test the IL-17 and IL-23 levels in plasma. Flow cytometry was used to detect the frequency of Th17 cells. The correlation between plasma IL-23 and IL-17 levels, Th17 cells, platelets were analyzed. The level of Th17-related cytokines was measured by ELISA following stimulation with IL-23. Subsequently, the IL-23 and IL-17 levels were measured in patients post-treatment.

**Results:**

The PBMCs of ITP patients showed increased mRNA expression levels in each of the following: IL-23p19, IL-12p40, IL-23R, IL-12Rβ1, IL-17A, IL-17F, and RORC. In addition, elevated Th17 cells and plasma IL-17, IL-23 levels were also observed in these ITP patients. Furthermore, it was found that IL-23 levels in plasma are positively correlated with IL-17 levels and Th17 cells, yet negatively correlated with platelet count. Following IL-23 stimulation *in vitro*, IL-17 levels showed significant elevation. Furthermore, both IL-23 and IL-17 levels decreased after effective treatment.

**Conclusion:**

The IL-23/Th17 pathway may be involved in the pathogenesis of ITP through enhancement of the Th17 response. Moreover, our results suggest that the IL-23/Th17 pathway is a potential therapeutic target in future attempts of ITP treatment.

## Introduction

Primary immune thrombocytopenia (ITP) is the most common hemorrhagic autoimmune disease, and is characterized by an isolated thrombocytopenia that is not accompanied by any other disorders that may lead to thrombocytopenia[1]. Recent studies have found that cellular immunity deficiency plays an important role in the pathogenesis of ITP, including B-cell activation, T-cell related disorders and antigen-presenting cell functional defect[2–4]. Significantly, it is well-recognized that an abnormal T-cell-mediated response is strongly correlated to the development and progress in ITP[5].

Recent studies have demonstrated that Th17, which is characterized for its production of IL-17, is elevated in ITP patients [6,7]. IL-17 belongs to the IL-17 cytokine family, which contains 6 different IL pro-inflammatory cytokines, from IL-17A to IL-17F. IL-17A is often represented as IL-17. The high degree of similarity between IL-17A and IL-17F is significant, and they also have a common biological function. Increased IL-17 expression has been observed in various autoimmune diseases, such as rheumatoid arthritis (RA)[8] and systemic lupus erythematosus(SLE)[9]. This evidence suggests that IL-17 may be associated with autoimmune diseases.

IL-23, which is mainly secreted by antigen-presenting cells, is a member of the IL-12 family, which includes IL-12, IL-27, and IL-35[10]. IL-23 is a heterodimeric cytokine, comprised a unique p19 subunit and p40 subunit, the latter of which is shared with IL-12. The receptor for IL-23 consists of IL-23R and IL-12Rβ1, the latter of which is also characteristic of IL-12. IL-23 is essential for Th17 differentiation, expansion, and survival by binding to its receptor, thereby activating the signaling pathway [11,12]. Many studies revealed that the IL-23/Th17 pathway is implicated in the pathophysiology of various autoimmune diseases, such as autoimmune arthritis[13], primary biliary cirrhosis[14], and inflammatory bowel disease[15]. But the important role of IL-23 in human ITP, especially in relation to Th17 cells remains unsettled.

In the present study, we used quantitative real-time PCR to investigate the gene expression of the subunits of IL-17, IL-23, and their receptors in ITP patients and healthy controls. Plasma levels of IL-17 and IL-23 were analyzed by ELISA. The frequency of Th17 cells were detected by flow cytometry. Moreover, we explore the function of IL-23 in Th17-associated cytokine production in ITP patients.

## Materials and Methods

### Patients

We enrolled 30 patients with confirmed acute ITP who were admitted to the Shanghai Changhai Hospital and Shanghai Tongji Hospital from June 2012 to January 2014. An ITP diagnosis was made according to a report from an international working group [16]. We excluded subjects who had the complications of diabetes, hypertension, cardiovascular diseases, pregnancy, active infection, or autoimmune diseases other than ITP [17]. We followed up on eleven newly diagnosed patients after effective treatment in order to analyze the dynamic change of IL-23. The criteria for assessing response to ITP treatments was according to a previous report [16].

As a control group, we also enrolled 30 healthy subjects (18 females and 12 males; age range: 19–62 years; median age: 36 years). All controls had normal platelet counts and did not receive any steroid therapy. There was no statistical difference between the control group and the ITP group in age and gender (P>0.05).

Our study was approved by the Ethics Committee of Shanghai Changhai Hospital and Shanghai Tongji Hospital (Shanghai, China). Written informed consent was obtained from all subjects.

### PBMC isolation and cell culture

Peripheral whole blood was obtained from all subjects. PBMC were isolated using Ficoll-Hypaque density gradient centrifugation. For experiments in vitro, PBMCs from ITP patients were cultured at a concentration of 1×10^6^ cells/mL in RPMI1640 medium (Sigma, R8758) supplemented with 10% fetal bovine serum, 100U/ml penicillin and 100mg/mL streptomycin (Invitrogen, USA) as mentioned previously[18], and stimulated with recombinant IL-23 (R&D Systems, 1290-IL-010) or PRMI1640. Culture supernatant was collected for cytokine analysis. For future flow cytometric analysis, PBMCs were cryopreserved in fetal bovine serum containing 10% dimethyl sufloxide (DMSO), and stored in liquid nitrogen.

### Total RNA extraction and cDNA synthesis

Total RNA was extracted using the RNeasy Mini Kit (Qiagen Inc, 74104) according to the manufacturer’s instruction. RNA yield and purity were determined spectrophotometrically at 260/280 nm and the ratios were in the range of 1.8–2.0. cDNA was synthesized using 1 μg total RNA with random primers and TaqMan Reverse Transcription Kit (ABI, N8080234), and stored at -20°C until use.

### Quantitative real-time PCR

All PCR reactions were undertaking on ABI7500. Primers and TaqMan-MGB probes were designed for detecting IL-23p19, IL-12p35, IL-12p40, IL-23R, IL-12Rβ1, IL-12Rβ2, and 18s-RNA mRNA expression as described[14], and 18s-RNA was used as internal control. The mRNA expression for IL-17A, and IL-17F, RORC took place using SYBR Green method, and GAPDH was used as internal control. The primers used were as follows: IL-17A forward 5’-ATGACTCCTGGGAAGACCTCATTG-3’; reverse 5’-TTAGGCCACATGGTGGACAATCGGG-3’; IL-17F forward 5’-GTCACTTGGGACCCCAACCGG-3’; reverse 5’-CTGCACATGGTGGATGACAG GG-3’; RORC forward 5’-GTCCCGAGATGCTGTCAAGT-3’; reverse 5’-TGAGGGTATCTGCTCCTTGG-3’; GAPDH forward5’-TGGTATCGTGGAAGGAC TCA-3’; reverse 5’-CCAGTAGAGGCAGGGATGAT-3’. All samples were tested in triplicate. The CT values of internal control and a target gene were determined. The relative expression for the target gene was given by 2^-ΔΔ CT^.

### Flow Cytometric Analysis

Cryopreserved PBMCs were thawed at 37°C, washed twice with PBS, and stained with trypan blue to determine cell viability. PBMCs were adjusted to a concentration of 1×10^6^/ml in RPMI1640 medium supplemented with 10% fetal bovine serum. The PBMCs were incubated for 4 hours at 37°C, 5% CO_2_ in the presence of PMA/Ionomycin mixture (Liankebio, LK-CS1001) and BFA/Monensin mixture (Liankebio, LK-CS1002) according to the manufacturer’s instructions. After incubation, the cells were stained with FITC-conjugated anti-CD3 (BD, USA) and PE-Cy5-conjugated anti-CD8 (Biolegend, 301010) to delimitate CD4^+^ T cells. Then the cells were stained with PE-conjugated anti-IL-17A (Biolegend, 512306) for Th17 detection after fixation and permeabilization (BD IntraSure Kit, 4043524). Stained cells were tested on a FACS Calibur flow cytometer (BD, USA) and then analyzed using CellQuest software (BD Bioscience).

### ELISA

IL-17 and IL-23 levels in plasma were detected using commercial enzyme-linked immunosorbent assay (ELISA) according to the manufacturer’s instructions. Concentrations of IL-6, IL-17, and IL-21 in culture supernatant were determined using Bio-Plex Pro Human Th17 cytokine assay (BIO-RAD).

### Statistical analysis

Statistical analysis was performed using GraphPad Prism 5.0 software (GraphPad Software Inc). Unpaired Student’s t test or Mann-Whitney U test was used, as appropriate, to compare results between groups. Correlations between variables were assessed by Pearson or Spearman correlation coefficients, as appropriate. A paired t-test was used to compare results before and after treatment. A p-value of <0.05 was considered significantly different.

## Result

### Characteristics of participants

Characteristics of the 30 ITP patients admitted in our study are shown in Table 1. The clinical information of 11 newly diagnosis ITP patients was shown in Table 2. Complete response was observed in 7, response in 4 of the 11 newly diagnosis patients. During the treatment, no bleeding or other obvious complications were observed.

**Table 1:** ITP patient’s clinical characteristics.


Patient No	Gender	Age/year	Course/month	Bleeding symptom	PLT/×10^9^/on admission	Previous Treatment
1	F	18	4	PT, EC	19	—
2	F	24	13	EP, EC	23	DXM
3	M	43	36	PT	14	DXM
4	F	26	20	PT, EC	8	Pred
5	M	44	28	GH	11	DXM
6	M	38	14	EC	16	DXM
7	F	61	7	PT	10	—
8	M	24	1	PT, EC	9	—
9	F	33	2	GH	7	—
10	M	41	7	PT, EC	14	—
11	F	70	31	EC, EP	11	Pred
12	M	51	26	EP	6	DXM
13	M	32	36	EP, EC	6	Pred
14	M	20	7	EP	12	—
15	F	34	6	EP	11	—
16	F	50	29	EC	4	DXM
17	M	27	30	PT	10	DXM
18	F	39	8	PT	22	—
19	F	28	41	EC, PT	14	DXM
20	F	22	22	PT, GH	27	Pred
21	M	25	5	EC, PT	21	—
22	F	19	1	GUH	11	—
23	F	26	31	EP	7	DXM
24	F	39	26	PT, EP	12	DXM
25	F	47	37	PT	10	Pred
26	F	41	21	PT	21	DXM
27	F	24	9	GUH	16	—
28	M	36	48	EP, PT	25	Pred
29	M	29	22	PT	7	DXM
30	F	46	14	PT	15	DXM

F:female; M: male; PT: petechiae; EC: ecchymoses; EP: epistaxis; GUH: genitourinary hemorrhage; GH: gingival hemorrhage;—:no prior treatment; PRED: prednisone; DXM: dexamethasone

**Table 2:** Eleven newly diagnosis ITP patients clinical information.

Patient No	Treatment	Dose(mg/day)	Period of treatment/day	PLT before treatment(×10^9^/L)	PLT after treatment(×10^9^/L)
1	DXM	40	4	19	52
7	DXM	40	4	10	125
8	DXM	40	4	9	76
9	DXM	40	4	7	154
10	DXM	40	4	14	37
14	DXM	40	4	12	63
15	DXM	40	4	11	151
18	DXM	40	4	22	257
21	DXM	40	4	21	105
22	DXM	40	4	11	127
27	DXM	40	4	16	172

DXM: dexamethasone

### Increased IL-23p19, IL-12p40, IL-23R, IL-12Rβ1, IL-17A, IL-17F, RORC mRNA expression in PBMCs from patients with primary immune thrombocytopenia

**Fig 1:**
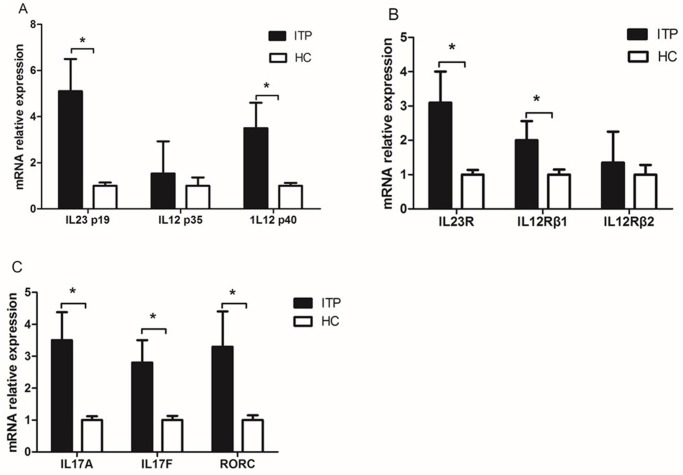
IL-23/Th17 pathway related molecules mRNA relative expression. *P<0.01.

As shown in Fig. 1, compared to healthy controls, IL-23 p19 and p40 mRNA expression were both significantly increased in ITP patients, but not IL-12 p35 mRNA (Fig. 1A). Moreover, we analyzed the expression of their relative receptors. We found IL-23R and IL-12Rβ1 mRNA expression increased, but not IL-12Rβ2 (Fig. 1B). The expression of Th17-related RORC, IL-17A and IL-17F also significantly increased (Fig. 1C). There was no statistical difference in these measures between newly diagnosis group and re-activated group.

### Increased plasma IL-23, IL-17 level and Th17 cells in patients with primary immune thrombocytopenia

**Fig 2:**
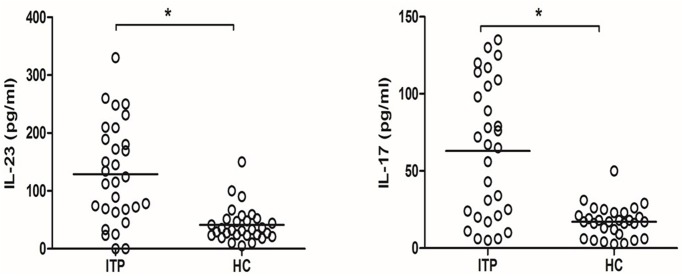
Plasma IL-23 and IL-17 levels in ITP patients and healthy controls. *P<0.01.

**Fig 3:**
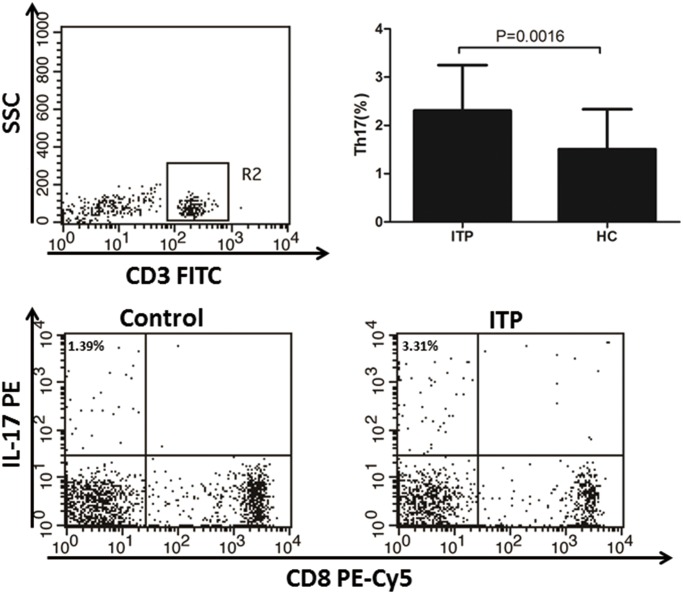
Percentage of Th17 cells in ITP patients and healthy controls.

Compared to healthy controls, the IL-23 and IL-17 level in plasma from ITP patients were both significantly increased (Fig. 2). We did not observed any statistical difference in plasma IL-23 and IL-17 levels between newly diagnosis group and re-activated group. In flow cytometry analysis, the percentage of Th17 cells in ITP patient was increased compared to that in healthy controls (Fig. 3).

### Correlation between plasma IL-23 and IL-17 level, Th17 cells, platelets

**Fig 4:**
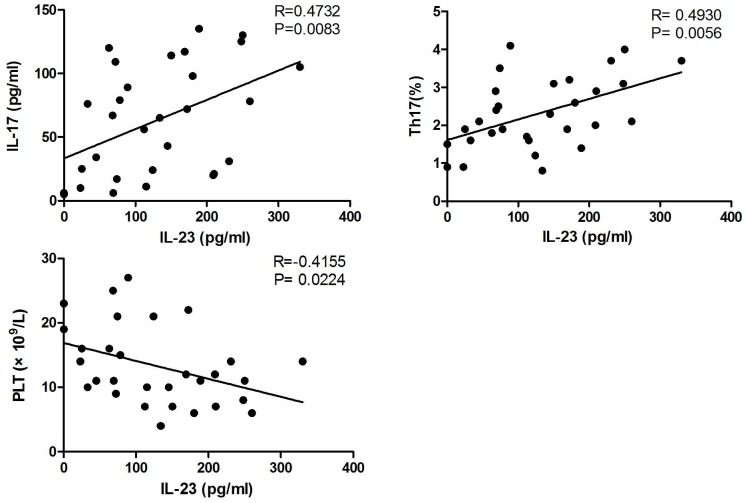
Correlation between plasma IL-23 and IL-17, Th17, platelets in ITP patients.

As shown in Fig. 4, IL-23 level in ITP plasma were positively correlated with plasma IL-17 and Th17 cells, while negatively related with platelet counts.

### Increased Th17 related cytokines by PBMC from ITP patients after IL-23 stimulation in vitro

**Fig 5:**
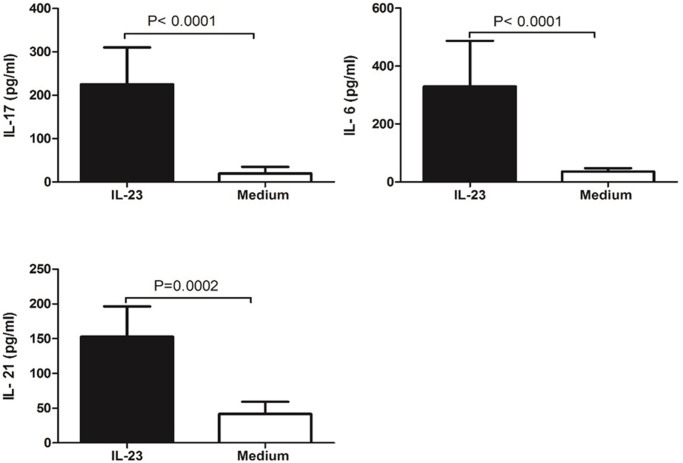
Th17 related cytokines production in culture supernatant in the presence and absence of IL-23.

In order to further validate the role of IL-23 on Th17 related-cytokine production induced by PBMC, we incubated the PBMC from ITP patients with rhIL-23 or medium (as control). We observed that the IL-6, IL-17, and IL-21 levels in culture supernatant increased after rhIL-23 stimulation (Fig. 5). The Th17 related cytokines production in newly diagnosis and re-activated groups were not reached statistical differences.

### Decreased plasma IL-23 level after effective treatment

**Fig 6:**
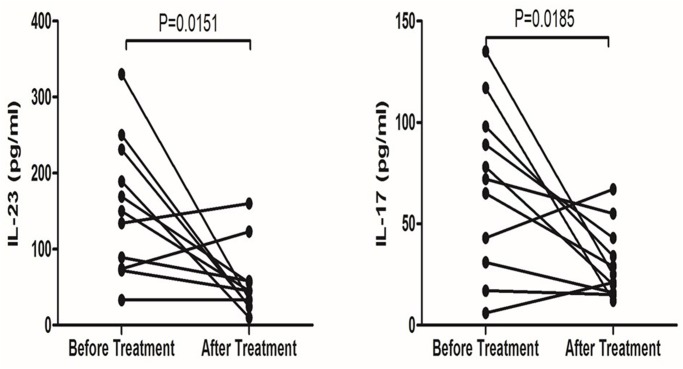
Plasma IL-23 and IL-17 level change in ITP patients after effective treatment.

We followed up eleven newly diagnosed ITP patients after admission to this study. We found that the plasma of these patients showed decreased levels of IL-23 and IL-17 after effective treatment (Fig. 6).

## Discussion

In the present study, we found that the mRNA expression of IL-23 subunits p19 and p40, IL-23 receptors, IL-17 and RORC is increased in patients with ITP. We also reported that plasma IL-17, IL-23 levels and Th17 cells were increased in ITP patients. A positive correlation exists between plasma IL-23 and IL-17 and Th17 levels. To the best of our knowledge, this is the first time that abnormal IL-23 expression and it’s correlation with elevated IL-17 were observed in ITP patients.

IL-23, which is known as a pro-inflammatory mediator, maintains the balance between regulatory and effector T-cells, and it is indispensable for promoting autoimmunity through T-cell-mediated inflammation [19–21]. Recently, numerous studies have found that increased expression of IL-23 is involved in the pathogenesis of many autoimmune diseases, such as inflammatory bowel disease, Crohn’s disease (CD)[15], and multiple sclerosis (MS)[22]. IL-23 exerts its biological effects by binding to its receptors. IL-23 receptors consist of IL-23R and IL-12Rβ1, while the receptor for IL-12 consists of the common IL-12Rβ1 and a unique IL-12Rβ2 [23]. Our results show that the IL-12 unique subunit p35 and IL-12Rβ2 were not elevated in ITP patients. Therefore, our results showed the increased expression of common IL-12p40 and receptor were caused by IL-23, and not IL-12. Previous study demonstrated that IL-23 can be induced by Toll-like receptor (TLR) agonists and interactions with T cells (CD40/CD40L interaction) [24]. Liu et al found that TLR4 was abnormal expression in ITP patients and demonstrated that TLR4 in combination with FcγRII could cause DCs to express cytokines IL-23 then promote effector Th17 cell responses [25]. These may suggest that the elevated of IL-23 may cause by abnormal TLR, but the exact mechanism need further study.

Th17, a newly defined CD4^+^ Th subset, has been implicated in the pathogenesis of many diseases, particularly autoimmune disorders [26,27]. Th17 modulates the pro-inflammatory response by producing IL-17, IL-6, IL-21 and other mediators [28,29]. Until now, many studies have revealed that Th17 cell was increased in ITP patients. Ji et al found that the ratio of Treg/Th17 correlated with the disease activity might have prognostic role in ITP [30]. Rocha et al found that increased levels of IL-17A and of Th17-related cytokines contribute to the pathogenesis of ITP [31]. Wang et al found that elevation of IL-17 and IFN-γ may be an important dysregulation of cellular immunity in pediatric patients with chronic ITP [32]. We found that IL-17 was increased both at the mRNA and protein levels in ITP patients, which is consistent with previous studies. RORC, the gene encoding Th17 transcription factor, RORγT, is a specific regulator for Th17 differentiation[33]. In the present study, we found that RORC mRNA expression is increased in ITP patients. These results indicate that Th17 is an abnormal activator in ITP patients. Although it was shown in previous studies [6,34], our work focused on the correlations between IL-23 and Th17 cells and related cytokines in ITP patients for the first time. This provides new insights on the pathogenesis mechanisms of Th17 in ITP.

Recent studies have shown that Th17 differentiation and activation is regulated by IL-23[12]. To date, IL-23 is known as the potent cytokine that promotes IL-17 production by Th17 cells. Although IL-6 and TGF-β can also drive the differentiation of Th17 cells from naïve CD4^+^ T cells [35], IL-23 is the key factor for the maturation and phenotype stabilization of pathogenic Th17 cells[12,36]. Th17 development was suspended in the early activation stage without IL-23[37]. IL-23 exerts its biological function via its receptor in order to induce Janus family kinase (Jak2) and tyrosine kinase 2 (Tyk2) phosphorylation, which is followed by the activation of STAT1, STAT3, STAT4 and STAT5[38–40]. Among these, the activation of STAT3 by IL-6 and IL-23 is crucial in Th17 lineage differentiation. Numerous studies clearly suggested that IL-23 promotes the pathogenicity of Th17 cells through several mechanisms: 1.) maintenance the expression of Th17 signature gene (RORC and IL-17); 2.) induction of effector genes (IL-22, Csf2 and IFN-γ) and down-regulation of repressive genes (IL-2, IL-27 and IL-12); 3.) amplification of its own signal through the up-regulation of IL-23R expression[41]. In the present study, we found a positive correlation between IL-23 and IL-17 levels in plasma from ITP patients, which suggests that IL-17 production is influenced by IL-23.

In order to investigate the role of IL-23 in IL-17 production in ITP, we cultured PBMCs from ITP patients with exogenous rhIL-23 *in vitro*. Our results showed increased IL-6, IL-17, and IL-21 expression in the culture supernatant following IL-23 stimulation. This suggests that IL-23 is involved in ITP through activation of the Th17 response. We also found a negative relationship between IL-23 levels and platelet counts. To some extent, the platelet counts reflect the disease severity as well as the development of the disease[42]. So IL-23 levels could serve as a potential index to evaluate the disease state.

Moreover, we followed up on 11 patients. Our results showed that both IL-17 and IL-23 decreased after effective treatment. It is widely accepted that glucocorticoids are the first-line treatment approach for ITP in clinic[43]. Several previous studies found that high-dose dexamethasone could correct the T cell subsets and cytokine profiles in ITP [44–46]. Moreover, Li et al [44] found that dexamethasone corrected the T cell subset levels by promoting GATA3 and FOXp3 expression and inhibiting RORC expression. In the present study, we found that IL-17 expression decreased after treatment. This result may be caused by the decreased number of Th17 cells after dexamethasone treatment through depression of RORC.

Based on the above results, we presume that the elevated IL-23 expression in the plasma of ITP patients promoted Th17 responses through RORC up-regulation and the STAT3 signal pathway. However, the underlying mechanism requires further investigation, and it is thereby a limitation in our study.

In conclusion, the IL-23/Th17 pathway is engaged in the development of ITP. Monitoring the IL-23 level in plasma may aid in evaluating the ITP disease state. Further studies may focus on the possibility of employing anti-IL-23 drugs to treat ITP patients.

## References

[1] McKenzie CG, Guo L, Freedman J, Semple JW (2013) Cellular immune dysfunction in immune thrombocytopenia (ITP). Br J Haematol 163: 10–23. 10.1111/bjh.12480 23937260

[2] Semple JW, Provan D, Garvey MB, Freedman J (2010) Recent progress in understanding the pathogenesis of immune thrombocytopenia. Curr Opin Hematol 17: 590–595. 10.1097/MOH.0b013e32833eaef3 20739879

[3] Coopamah MD, Garvey MB, Freedman J, Semple JW (2003) Cellular immune mechanisms in autoimmune thrombocytopenic purpura: An update. Transfus Med Rev 17: 69–80. 12522773 10.1053/tmrv.2003.50004

[4] Cooper N, Bussel J (2006) The pathogenesis of immune thrombocytopaenic purpura. Br J Haematol 133: 364–374. 16643442 10.1111/j.1365-2141.2006.06024.x

[5] Semple JW, Provan D (2012) The immunopathogenesis of immune thrombocytopenia: T cells still take center-stage. Curr Opin Hematol 19: 357–362. 10.1097/MOH.0b013e3283567541 22759631

[6] Hu Y, Li H, Zhang L, Shan B, Xu X, et al (2012) Elevated profiles of Th22 cells and correlations with Th17 cells in patients with immune thrombocytopenia. Hum Immunol 73: 629–635. 10.1016/j.humimm.2012.04.015 22537755

[7] Huber M, Heink S, Grothe H, Guralnik A, Reinhard K, et al (2009) A Th17-like developmental process leads to CD8(+) Tc17 cells with reduced cytotoxic activity. Eur J Immunol 39: 1716–1725. 10.1002/eji.200939412 19544308

[8] Roeleveld DM, van Nieuwenhuijze AE, van den Berg WB, Koenders MI (2013) The Th17 pathway as a therapeutic target in rheumatoid arthritis and other autoimmune and inflammatory disorders. BioDrugs 27: 439–452. 10.1007/s40259-013-0035-4 23620106

[9] Ballantine LE, Ong J, Midgley A, Watson L, Flanagan BF, et al (2014) The pro-inflammatory potential of T cells in juvenile-onset systemic lupus erythematosus. Pediatr Rheumatol Online J 12: 4 10.1186/1546-0096-12-4 24433387 PMC3898918

[10] Gee K, Guzzo C, Che Mat NF, Ma W, Kumar A (2009) The IL-12 family of cytokines in infection, inflammation and autoimmune disorders. Inflamm Allergy Drug Targets 8: 40–52. 19275692 10.2174/187152809787582507

[11] Toussirot E (2012) The IL23/Th17 pathway as a therapeutic target in chronic inflammatory diseases. Inflamm Allergy Drug Targets 11: 159–168. 22280236 10.2174/187152812800392805

[12] Langrish CL, Chen Y, Blumenschein WM, Mattson J, Basham B, et al (2005) IL-23 drives a pathogenic T cell population that induces autoimmune inflammation. J Exp Med 201: 233–240. 15657292 10.1084/jem.20041257 PMC2212798

[13] Lubberts E (2008) IL-17/Th17 targeting: on the road to prevent chronic destructive arthritis? Cytokine 41: 84–91. 18039580 10.1016/j.cyto.2007.09.014

[14] Qian C, Jiang T, Zhang W, Ren C, Wang Q, et al (2013) Increased IL-23 and IL-17 expression by peripheral blood cells of patients with primary biliary cirrhosis. Cytokine 64: 172–180. 10.1016/j.cyto.2013.07.005 23910013

[15] Fransen K, van Sommeren S, Westra HJ, Veenstra M, Lamberts LE, et al (2014) Correlation of genetic risk and messenger RNA expression in a Th17/IL23 pathway analysis in inflammatory bowel disease. Inflamm Bowel Dis 20: 777–782. 10.1097/MIB.0000000000000013 24662057

[16] Rodeghiero F, Stasi R, Gernsheimer T, Michel M, Provan D, et al (2009) Standardization of terminology, definitions and outcome criteria in immune thrombocytopenic purpura of adults and children: report from an international working group. Blood 113: 2386–2393. 10.1182/blood-2008-07-162503 19005182

[17] Liu XG, Ma SH, Sun JZ, Ren J, Shi Y, et al (2011) High-dose dexamethasone shifts the balance of stimulatory and inhibitory Fcgamma receptors on monocytes in patients with primary immune thrombocytopenia. Blood 117: 2061–2069. 10.1182/blood-2010-07-295477 21131591

[18] Kobayashi T, Okamoto S, Hisamatsu T, Kamada N, Chinen H, et al (2008) IL23 differentially regulates the Th1/Th17 balance in ulcerative colitis and Crohn’s disease. Gut 57: 1682–1689. 10.1136/gut.2007.135053 18653729

[19] Izcue A, Hue S, Buonocore S, Arancibia-Carcamo CV, Ahern PP, et al (2008) Interleukin-23 restrains regulatory T cell activity to drive T cell-dependent colitis. Immunity 28: 559–570. 10.1016/j.immuni.2008.02.019 18400195 PMC2292821

[20] Cua DJ, Sherlock J, Chen Y, Murphy CA, Joyce B, et al (2003) Interleukin-23 rather than interleukin-12 is the critical cytokine for autoimmune inflammation of the brain. Nature 421: 744–748. 12610626 10.1038/nature01355

[21] Kastelein RA, Hunter CA, Cua DJ (2007) Discovery and biology of IL-23 and IL-27: related but functionally distinct regulators of inflammation. Annu Rev Immunol 25: 221–242. 17291186 10.1146/annurev.immunol.22.012703.104758

[22] Romme Christensen J, Bornsen L, Hesse D, Krakauer M, Sorensen PS, et al (2012) Cellular sources of dysregulated cytokines in relapsing-remitting multiple sclerosis. J Neuroinflammation 9: 215 10.1186/1742-2094-9-215 22978757 PMC3503813

[23] Paradowska-Gorycka A, Grzybowska-Kowalczyk A, Wojtecka-Lukasik E, Maslinski S (2010) IL-23 in the pathogenesis of rheumatoid arthritis. Scand J Immunol 71: 134–145. 10.1111/j.1365-3083.2009.02361.x 20415779

[24] Brentano F, Ospelt C, Stanczyk J, Gay RE, Gay S, et al (2009) Abundant expression of the interleukin (IL)23 subunit p19, but low levels of bioactive IL23 in the rheumatoid synovium: differential expression and Toll-like receptor-(TLR) dependent regulation of the IL23 subunits, p19 and p40, in rheumatoid arthritis. Ann Rheum Dis 68: 143–150. 10.1136/ard.2007.082081 18276743

[25] Liu H, Ouyang X, Li Y, Zeng H, Wang X, et al (2013) Involvement of levels of Toll like receptor-4 in monocytes, CD4+ T-lymphocyte subsets, and cytokines in patients with immune thrombocytopenic purpura. Thromb Res 132: 196–201. 10.1016/j.thromres.2013.04.025 23830211

[26] Noack M, Miossec P (2014) Th17 and regulatory T cell balance in autoimmune and inflammatory diseases. Autoimmun Rev 13: 668–677. 10.1016/j.autrev.2013.12.004 24418308

[27] Wang X, Zheng XY, Ma C, Wang XK, Wu J, et al (2014) Mitigated Tregs and augmented Th17 cells and cytokines are associated with severity of experimental autoimmune neuritis. Scand J Immunol. 10.1111/sji.12260 24910360

[28] Pappu R, Ramirez-Carrozzi V, Sambandam A (2011) The interleukin-17 cytokine family: critical players in host defence and inflammatory diseases. Immunology 134: 8–16. 10.1111/j.1365-2567.2011.03465.x 21726218 PMC3173690

[29] Romagnani S (2008) Human Th17 cells. Arthritis Res Ther 10: 206 10.1186/ar2392 18466633 PMC2453756

[30] Ji L, Zhan Y, Hua F, Li F, Zou S, et al (2012) The ratio of Treg/Th17 cells correlates with the disease activity of primary immune thrombocytopenia. PLoS One 7: e50909 10.1371/journal.pone.0050909 23226546 PMC3513316

[31] Rocha AM, Souza C, Rocha GA, de Melo FF, Clementino NC, et al (2011) The levels of IL-17A and of the cytokines involved in Th17 cell commitment are increased in patients with chronic immune thrombocytopenia. Haematologica 96: 1560–1564. 10.3324/haematol.2011.046417 21972211 PMC3186321

[32] Wang JD, Chang TK, Lin HK, Huang FL, Wang CJ, et al (2011) Reduced expression of transforming growth factor-beta1 and correlated elevation of interleukin-17 and interferon-gamma in pediatric patients with chronic primary immune thrombocytopenia (ITP). Pediatr Blood Cancer 57: 636–640. 10.1002/pbc.22984 21721104

[33] Mottaghi A, Ebrahimof S, Angoorani P, Saboor-Yaraghi AA (2014) Vitamin A supplementation reduces IL-17 and RORc gene expression in atherosclerotic patients. Scand J Immunol. 10.1111/sji.12260 24845870

[34] Zhu X, Ma D, Zhang J, Peng J, Qu X, et al (2010) Elevated interleukin-21 correlated to Th17 and Th1 cells in patients with immune thrombocytopenia. J Clin Immunol 30: 253–259. 10.1007/s10875-009-9353-1 19997967

[35] Bettelli E, Carrier Y, Gao W, Korn T, Strom TB, et al (2006) Reciprocal developmental pathways for the generation of pathogenic effector TH17 and regulatory T cells. Nature 441: 235–238. 16648838 10.1038/nature04753

[36] Zhou L, Ivanov II, Spolski R, Min R, Shenderov K, et al (2007) IL-6 programs T(H)-17 cell differentiation by promoting sequential engagement of the IL-21 and IL-23 pathways. Nat Immunol 8: 967–974. 17581537 10.1038/ni1488

[37] McGeachy MJ, Chen Y, Tato CM, Laurence A, Joyce-Shaikh B, et al (2009) The interleukin 23 receptor is essential for the terminal differentiation of interleukin 17-producing effector T helper cells in vivo. Nat Immunol 10: 314–324. 10.1038/ni.1698 19182808 PMC2945605

[38] Parham C, Chirica M, Timans J, Vaisberg E, Travis M, et al (2002) A receptor for the heterodimeric cytokine IL-23 is composed of IL-12Rbeta1 and a novel cytokine receptor subunit, IL-23R. J Immunol 168: 5699–5708. 12023369 10.4049/jimmunol.168.11.5699

[39] Fahey AJ, Robins RA, Kindle KB, Heery DM, Constantinescu CS (2006) Effects of glucocorticoids on STAT4 activation in human T cells are stimulus-dependent. J Leukoc Biol 80: 133–144. 16670125 10.1189/jlb.0605296

[40] Yang XO, Panopoulos AD, Nurieva R, Chang SH, Wang D, et al (2007) STAT3 regulates cytokine-mediated generation of inflammatory helper T cells. J Biol Chem 282: 9358–9363. 17277312 10.1074/jbc.C600321200

[41] Gaffen SL, Jain R, Garg AV, Cua DJ (2014) The IL-23-IL-17 immune axis: from mechanisms to therapeutic testing. Nat Rev Immunol 14: 585–600. 10.1038/nri3707 25145755 PMC4281037

[42] Kuhne T (2013) Update on the Intercontinental Cooperative ITP Study Group (ICIS) and on the Pediatric and Adult Registry on Chronic ITP (PARC ITP). Pediatr Blood Cancer 60 Suppl 1: S15–18. 10.1002/pbc.24342 23109493

[43] Gómez-Almaguer D, Tarín-Arzaga L, Moreno-Jaime B, Jaime-Pérez JC, Ceballos-López AA, et al (2013) High response rate to low-dose rituximab plus high-dose dexamethasone as frontline therapy in adult patients with primary immune thrombocytopenia. European Journal of Haematology 90: 494–500. 10.1111/ejh.12102 23470153

[44] Li J, Wang Z, Hu S, Zhao X, Cao L (2013) Correction of abnormal T cell subsets by high-dose dexamethasone in patients with chronic idiopathic thrombocytopenic purpura. Immunol Lett 154: 42–48. 10.1016/j.imlet.2013.08.006 23994430

[45] Zhan Y, Zou S, Hua F, Li F, Ji L, et al (2014) High-dose dexamethasone modulates serum cytokine profile in patients with primary immune thrombocytopenia. Immunol Lett 160: 33–38. 10.1016/j.imlet.2014.03.002 24657323

[46] Guo C, Chu X, Shi Y, He W, Li L, et al (2007) Correction of Th1-dominant cytokine profiles by high-dose dexamethasone in patients with chronic idiopathic thrombocytopenic purpura. J Clin Immunol 27: 557–562. 17619126 10.1007/s10875-007-9111-1

